# Environmental sampling for the detection of capripox viruses and peste des petits ruminants virus in households and livestock markets in Plateau State, Nigeria

**DOI:** 10.1099/acmi.0.000872.v3

**Published:** 2024-10-18

**Authors:** Emma Brown, David Ehizibolo, Banenat B. Dogonyaro, Yiltawe Wungak, Olumuyiwa Oyekan, Adeyinka Adedeji, Sandra Ijeoma, Rebecca Atai, Moses Oguche, Mark Samson, Fabrizio Rosso, Anna B. Ludi, Georgina Limon, Andrew E. Shaw, Claire Colenutt, Simon Gubbins

**Affiliations:** 1 The Pirbright Institute, Ash Road, Pirbright, Surrey, UK; 2 National Veterinary Research Institute, Vom, Plateau State, Nigeria; 3 European Commission for the Control of Foot-and-Mouth Disease, Food and Agriculture Organization of the United Nations, Rome, Italy

**Keywords:** disease surveillance, environmental sampling, livestock, transboundary animal disease

## Abstract

Multiple transboundary animal diseases (TADs) circulate in Plateau State, Nigeria, where livestock keeping is common and contributes to both the physical and socio-economic well-being of a large proportion of the population. In this study, we explored the potential for environmental sampling to detect viruses causing TADs circulating in the region. Electrostatic dust cloths were used to swab areas of the environment likely to have contact with secretions and excretions from infected animals. Samples were collected monthly from five households, one transhumance site and one livestock market in two local government areas in Plateau State between March and October 2021. These were tested for the presence of peste des petits ruminants virus (PPRV) and capripox viruses using real-time PCR. Of the 458 samples collected, 2.4% (*n* = 11) were positive for PPRV RNA and 1.3 % (*n* = 6) were positive for capripox virus DNA. A capripox differentiation assay showed that these samples were positive for sheep pox virus (*n* = 2), goat pox virus (*n* = 2) and lumpy skin disease virus (*n* = 2). Our results demonstrate that environmental sampling could be used as part of TAD surveillance in the area. Environmental swabs require little technical knowledge to collect and can be used to detect multiple viruses from a single sample.

## Data Summary

All data on the samples and test results are provided in the supplementary material, available in the online version of this article.

## Introduction

In Nigeria, the agriculture sector is an important source of livelihood and 42% of the population live in households that own livestock [1]. Transboundary animal diseases (TADs) are a socio-economic burden, particularly in regions where the practice of keeping livestock contributes significantly to physical and economic well-being [2]. The importance of healthy livestock is critical, as the presence of TADs can have a direct impact on food supply, reduce production and generate additional costs due to prevention and control measures.

Peste des petits ruminants virus (PPRV) [3] and capripox viruses, including lumpy skin disease virus (LSDV), goat pox virus (GTPV) and sheep pox virus (SPPV) [4,5] are four viruses endemic in Nigeria that cause TADs. Between 2020 and 2023, thousands of cases of peste des petits ruminants were reported each year to the World Organisation for Animal Health (WOAH), around 10% of which had died [6]. The disease was estimated to cost over US $10 million per annum at a national level [7] and has been estimated to cause losses of over 10% of a household’s annual income [8]. Over the same time period, hundreds of cases of goatpox, sheeppox and lumpy skin disease were reported each year to WOAH [6]. Costs for these diseases were estimated to be around US $30 million per annum at a regional level [9] and up to US $6340 at a household level [10].

Transmission routes for PPRV, LSDV, GTPV and SPPV vary, but they include close contact with infected animals, which is facilitated by the movement of infected animals, as well as through the bites of vectors in the case of LSDV [11,12]. All these viruses can be shed into the environment through secretions and excretions from infected livestock [12,13]. Only limited data are available on their stability in the environment, although capripox viruses are considered to be very stable and may survive for several months [12,14], while PPRV is likely to survive for a few days at most [11,15]. In addition, environmental sampling has been used previously to detect PPRV in the field [16] and LSDV in challenge experiments [17].

To gain insight into the occurrence and impact of TADs in Nigeria, reliable and convenient methods for surveillance must be available. In developing countries, veterinary resources are often insufficient to facilitate effective prevention and control of livestock diseases. A considerable part of this is the lack of effective surveillance, which can be resource-intensive. In this study, we explore the potential use of environmental sampling as a non-invasive, herd-level sampling approach in the surveillance of TADs. As sampling the environment is non-disease specific and independent of clinical observation, this sampling approach could be used for the detection of multiple viruses that cause common TADs. In this study, we used environmental samples previously tested for foot-and-mouth disease virus (FMDV) [18] to investigate the use of molecular methods to detect LSDV, GTPV, SPPV and PPRV in environmental samples collected from livestock markets, transhumance sites and households keeping livestock in Nigeria.

## Methods

### Study period and location

**Fig. 1.**
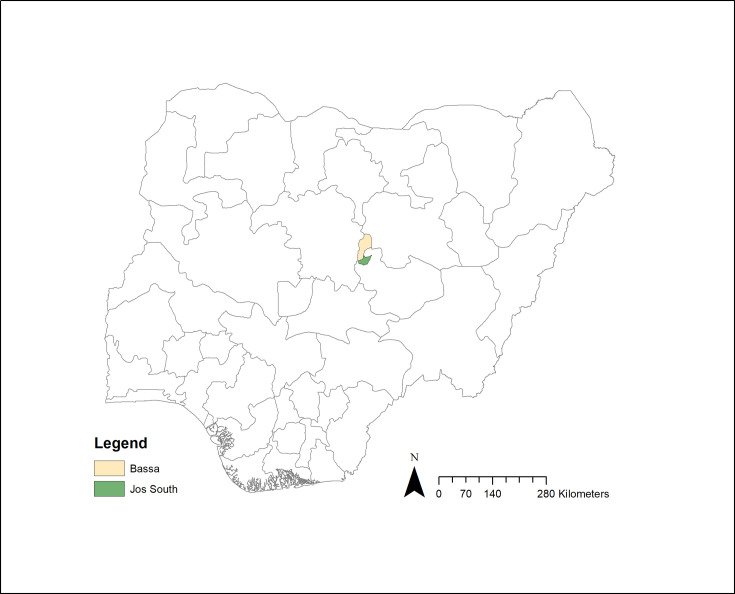
Map showing the location of Bassa and Jos South local government areas in Plateau State, Nigeria where environmental sampling was carried out between May and October 2021.

**Fig. 2.**
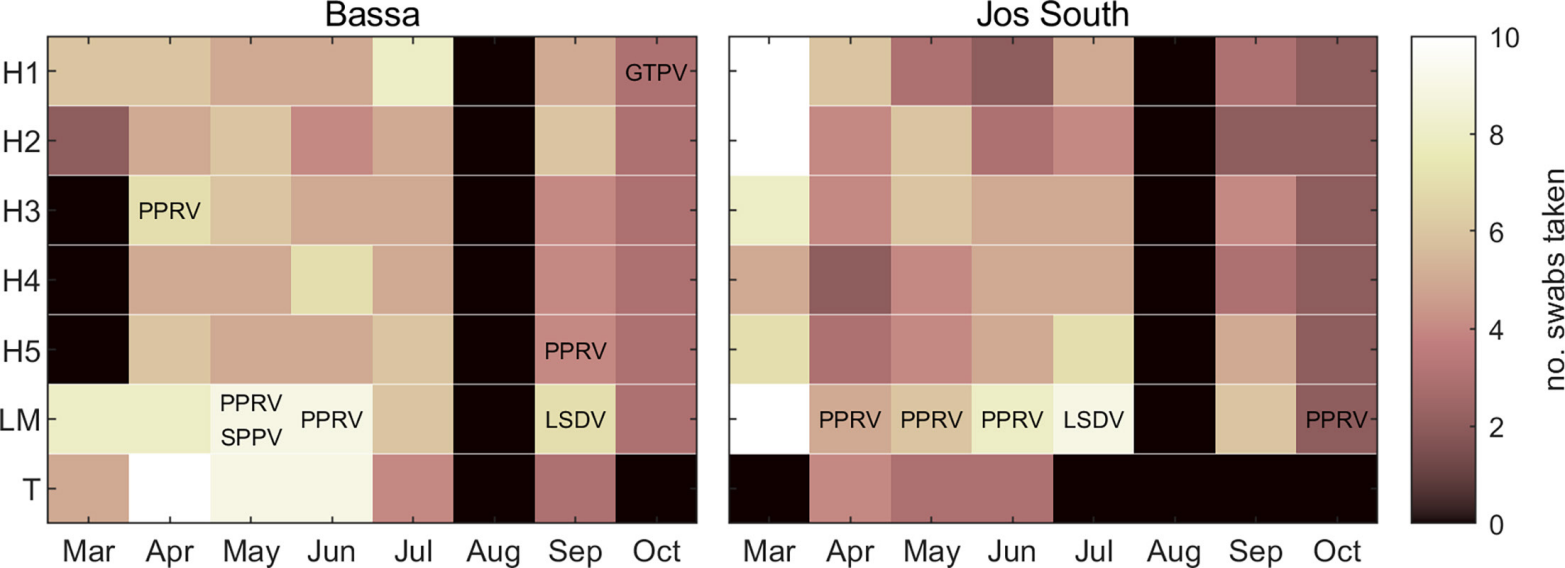
Longitudinal environmental sampling for the presence of PPRV, SPPV, GTPV and LSDV in Plateau State, Nigeria, between March and October 2021. Samples were collected from five households (**H1–H5**), one livestock market (LM) and one transhumance location (**T**) in both Bassa and Jos South local government areas. Coloured shading indicates the number of swabs taken (see colour bar). The virus name indicates a sampling occasion on which that virus was detected in at least one of the swabs (see Table 1 for full details).

Samples used in this study were collected as part of a longitudinal study of FMDV in Plateau State, Nigeria [18]. Two local government areas (LGAs) within Plateau State, Bassa and Jos South (Fig. 1), were the focus of sampling based on their high risk of foot-and-mouth disease [19]. Within each LGA, five households, one livestock market and one transhumance site were selected. Households were identified using local contacts and had to keep cattle and sheep/goats and agree to participate in the study. There is only a single livestock market and a single transhumance site in Jos South, while the livestock market and transhumance site in Bassa were selected based on access and agreement from those in charge to take part in the study. Each location was sampled once a month between March 2021 and October 2021 (Fig. 2), although sampling was not possible in August 2021 for security reasons.

### Environmental sampling

**Fig. 3.**
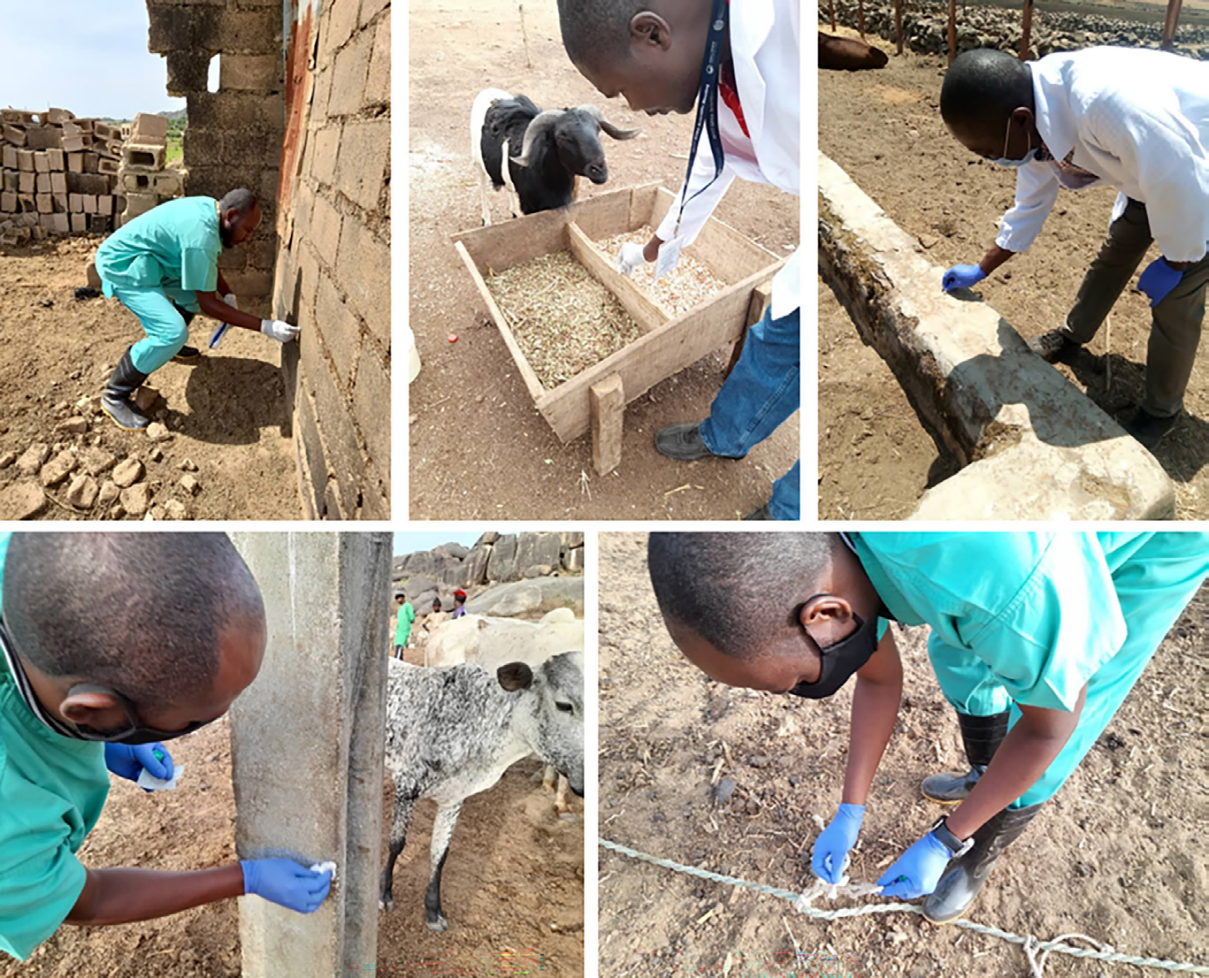
Examples of collection of swabs from environmental surfaces at livestock dwellings, including swabs from walls, feed troughs and ropes.

At each sampling site, electrostatic dust cloths were used to swab areas of the environment where contact with secretions and excretions from infected animals was deemed likely (e.g. food troughs, hard floor surfaces, boots, tether ropes, transport vehicles and herder’s sticks) (Fig. 3). Up to ten environmental samples per site per visit were collected (Fig. 2). Each environmental sample was given a unique ID number, which was linked to the site, place from which the sample was collected and month of the visit. The environmental samples were processed in the field by eluting the swabs in 5 ml PBS and then adding the samples directly to lysis buffer (MagMAX Core or RLT buffer, Thermo Fisher Scientific, UK) at a ratio of 1 : 2.6. All samples were stored and transported at 4 °C to The Pirbright Institute, UK for processing.

### Sample processing

Nucleic acid extraction from samples was performed using the KingFisher Flex automated extraction platform (Thermo Fisher Scientific, UK), with the MagMAX CORE Nucleic Acid Purification Kit (Thermo Fisher Scientific, UK). Environmental samples were tested by PCR for detection of PPRV, SPPV, GTPV and LSDV, all of which are known to be circulating in the region. Specifically, rRT-PCR was used to detect the F-gene of the PPRV genome as previously described [20]. A pan-species rPCR [21] was used to detect capripox virus DNA, followed by a species-specific capripox differentiation assay. The differentiation assay targeted species-specific molecular markers in the G-protein-coupled chemokine receptor gene of the target sequence and used dual hybridization probes for their detection and quantitation using a melting curve analysis [22].

## Results

A total of 372 environmental samples were collected between March and July and a further 86 were collected in September and October (Fig. 2). Eleven (out of 458; 2.4%) samples were positive for PPRV RNA (C_T_ value range: 30.8–38.3) (Table 1; Fig. 2). Three were collected at the livestock market in Bassa during May (*n* = 2) and June (*n* = 1). Six were collected at the livestock market in Jos South during April (*n* = 1), May (*n* = 1), June (*n* = 3) and October (*n* = 1). Two were collected at different households sampled in April (*n* = 1) and September (*n* = 1) in Bassa. Positive samples were collected from herders’ sticks (*n* = 1), feed troughs (*n* = 1), transport vehicles (*n* = 1), ropes (*n* = 4), pegs (*n* = 1), hard surface floors (*n* = 1) and water troughs (*n* = 2).

**Table 1.** Detection of PPRV RNA and capripox viral DNA in environmental samples from Plateau State, Nigeria

Month	Location	Virus	C_T_ value*	Surface type
April	Bassa – Household (H3)	PPRV	36.81	Herder’s sticks
April	Jos South – Livestock market	PPRV	32.91	Feed trough
May	Jos South – Livestock market	PPRV	31.66	Transport vehicle
May	Bassa – Livestock market	PPRV/SPPV	34.22/38.39	Ropes
May	Bassa – Livestock market	PPRV/SPPV	30.79/38.76	Ropes
June	Jos South – Livestock market	PPRV	38.25	Water trough
June	Jos South – Livestock market	PPRV	37.71	Ropes
June	Jos South – Livestock market	PPRV	36.46	Pegs
June	Bassa – Livestock market	PPRV	31.75	Ropes
September	Bassa – Household (H5)	PPRV	31.03	Hard floor surface
October	Jos South – Livestock market	PPRV	34.36	Water trough
July	Jos South – Livestock market	LSDV	35.92	Transport vehicle
September	Bassa – Livestock market	LSDV	39.00	Rope
October	Bassa – Household (H1)	GTPV	40.18	Herder’s sticks
October	Bassa – Household (H1)	GTPV	34.62	Rope

N.B. The same livestock market in both Bassa and Jos South was sampled monthly throughout the study.

*C_T_ values for capripox DNA were obtained using a pan-species rPCR assay. A differentiation assay was used to determine specific species: goat pox virus (GTPV), sheep pox virus (SPPV) or (LSDV).

A total of six samples (out of 458; 1.3%) were positive for capripox viral DNA (C_T_ value range: 34.4–40.2) (Table 1; Fig. 2). Two were positive for SPPV, collected from ropes at the livestock market in Bassa in May. Two were positive for GTPV, collected from a herder’s stick and rope at one household in Bassa in October. Two were positive for LSDV, one collected from a transport vehicle at the livestock market in Jos South in July and the other collected from rope at the livestock market in Bassa in September.

The two samples that were positive for SPPV DNA were also positive for PPRV RNA (Table 1; Fig. 2). None of the other positive samples were positive for nucleic acid from more than one virus (FMDV [18], PPRV, GTPV or LSDV).

## Discussion

The pathogens for which we tested samples in this study were selected based on TADs that are endemic to the study area [3,5] and are known to display some level of persistence in the environment [11,12, 14, 15]. These viruses may not remain viable once shed by an infectious individual [11], but genetic material can remain detectable via molecular methods for variable time periods depending on the individual virus and environmental conditions [14,15]. Consequently, molecular detection will not provide information on the viability of the detected virus, but as this study was focused on the detection of environmental contamination rather than assessing the potential for onward transmission, this is not necessarily a limitation. Further characterization of detected viruses, for example, using next-generation sequencing (NGS) [18,23], can provide additional valuable information for surveillance and control programmes. Developments in sequencing assays, such as the incorporation of a probe enrichment step in NGS library workflow, have enabled recovery of viral sequence data from environmental samples [18,23], which often contain poor quality or degraded genetic material, increasing the value of these sample types.

Environmental sampling has potential limitations in its application to surveillance. First, contamination of the samples with organic matter could affect the detection of a pathogen, for example by inhibiting the PCR reaction. However, we have not found this to be a problem for the sample types collected and the processing methods used in the present or previous studies [16,18, 23, 24]. Second, the detection of a pathogen in an environmental sample cannot easily be linked to a specific time and location of infection in animals in the same way that it can for surveillance in animals (e.g. clinical inspection, virological or serological sampling). However, it could be used as part of initial screening to inform risk-based surveillance optimizing resource allocation. Furthermore, the benefits in terms of increasing the accessibility of surveillance in resource-limited regions should be recognized. Environmental sampling can be enhanced by collecting further information from sampling sites, such as clinical observations and details of livestock movements, particularly for livestock markets, in order to provide context to the detection of viruses in the environment. However, this can be time consuming to collect if such information is not recorded routinely.

A third potential limitation is the overall proportion of positive environmental samples. In the present study, this was low for all four viruses (PPRV: 2.4%, 11/458; GTPV, SGPV and LSDV: 0.43%, 2/458). For each sampling time and location, however, only between two and ten swabs were taken, with 11–67% of swabs positive for viral nucleic acid when it was detected. This suggests that when and where samples are taken must be considered carefully. In the present study, most positive samples were found at livestock markets rather than households and, furthermore, were found at multiple time points at both markets (Fig. 2). Consequently, taking environmental samples regularly at livestock markets may be more efficient than taking them at households.

Because we used samples collected previously as part of an FMDV study [18], the sampling locations were selected based on the risk of foot-and-mouth disease [19] rather than on the risk of peste des petits ruminants or capripox diseases. For example, the risk of sheeppox and goatpox is higher in areas further to the north of those sampled in the present study [25]. A next step in assessing the utility of environmental sampling for PPRV, GTPV, SPPV or LSDV would be to design a study in high-risk areas for the disease they cause, collecting samples from animals and from the environment at the same locations, as we have done previously for FMDV [18]. This will help to understand how results for environmental samples relate to virus circulation in animals.

This study has shown that environmental swabs can potentially be used for the detection of multiple TADs in an endemic region, with viruses detected at both households keeping livestock and livestock markets. Given the frequency with which we detected viruses at them, perhaps the most immediately useful approach would be to take regular environmental swabs at livestock markets to identify and characterize viruses circulating in a market’s catchment area. This is likely to be valuable for integrating control of TADs [26] and during eradication campaigns, for example, as is planned for PPRV [27].

The role of environmental sampling in wider surveillance for TADs requires further assessment, especially of the relationship between results from sampling the environment and those from sampling animals. However, the collection of environmental swabs requires little technical skill or clinical knowledge, making this type of surveillance more accessible at the point of sample collection. Large areas and multiple sites can be sampled without compromising animal welfare or veterinarians’ security, and using minimal resources, potentially making this method more appealing and less time-consuming.

## Conclusions

Environmental sampling provides a convenient sampling technique for surveillance of TADs, which can improve knowledge of livestock viruses present in a specific region. It requires little technical knowledge and is not disease specific, so can be used to test for the presence of multiple pathogens in a single sample. In a situation where surveillance options are limited, environmental sampling presents a basic, low-cost option for introducing or complementing disease surveillance approaches in order to improve knowledge of circulating pathogens.

## supplementary material

10.1099/acmi.0.000872.v3 Uncited Supplementary Data Sheet 1.

## Abbreviations

FMDV: foot-and-mouth disease virus
GTPV: goat pox virus
LGAs: local government areas
LM: livestock market
LSDV: lumpy skin disease virus
NGS: next-generation sequencing
PPRV: peste des petits ruminants virus
SPPV: sheep pox virus
TADs: transboundary animal diseases
WOAH: World Organisation for Animal Health
